# New Insight for Axillary De-Escalation in Breast Cancer Surgery: “SoFT Study” Retrospective Analysis

**DOI:** 10.3390/curroncol31080309

**Published:** 2024-07-23

**Authors:** Gianluca Vanni, Marco Materazzo, Floriana Paduano, Marco Pellicciaro, Giordana Di Mauro, Enrica Toscano, Federico Tacconi, Benedetto Longo, Valerio Cervelli, Massimiliano Berretta, Oreste Claudio Buonomo

**Affiliations:** 1 Breast Unit, Department of Surgical Science, University of Rome “Tor Vergata”, Viale Oxford 81, 00133 Rome, Italy; vanni_gianluca@yahoo.it (G.V.); florianapaduano@gmail.com (F.P.); marco.pellicciaro@ptvonline.it (M.P.); benedetto.longo@uniroma2.it (B.L.); o.buonomo@inwind.it (O.C.B.); 2 Ph.D. Program in Applied Medical-Surgical Sciences, Department of Surgical Science, University of Rome “Tor Vergata”, Viale Oxford 81, 00133 Rome, Italy; 3 Department of Human Pathology “G. Barresi”, University of Messina, 98125 Messina, Italy; giordana.di.mauro@hotmail.it (G.D.M.); enrica.toscanoi@studenti.unime.it (E.T.); 4 Department of Surgical Sciences, Unit of Thoracic Surgery, Tor Vergata University, 00133 Rome, Italy; federico.tacconi@ptvonline.it; 5 Plastic and Reconstructive Surgery at Department of Surgical Science, Tor Vergata University of Rome, Via Montpellier 1, 00133 Rome, Italy; valeriocervelli@libero.it; 6 Department of Clinical and Experimental Medicine, University of Messina, 98100 Messina, Italy; berrettama@gmail.com; 7 General Surgery Program, Department of Health Science, UNIBAS, University of Basilicata, Via dell’Ateneo Lucano, 10, 85100 Potenza, Italy

**Keywords:** breast neoplasm, sentinel lymph node biopsy, axillary surgery, surgical de-escalation, early breast cancer

## Abstract

**Background**: The SOUND study demonstrated that an axillary de-escalation may be sufficient in locoregional and distant disease control in selected early breast cancer (EBC) patients. To establish any preoperative variables that may drive sentinel lymph node biopsy (SLNB) omission, a study named sentinel omission risk factor (SOFT) 1.23 was planned. **Methods:** A single-center retrospective study from a prospectively maintained database was designed, aiming at underlying preoperative prognostic factors involved in sentinel lymph node (SLN) metastasis (lymph node involvement (LN+) vs. negative lymph node (LN−) group). Secondary outcomes included surgical room occupancy analysis for SLNB in patients fulfilling the SOUND study inclusion criteria. The institutional ethical committee Area Territoriale Lazio 2 approved the study (n° 122/23). **Results:** Between 1 January 2022 and 30 June 2023, 160 patients were included in the study and 26 (%) were included in the LN+ group. Multifocality, higher cT stage, and larger tumor diameter were reported in the LN+ group (*p* = 0.020, *p* = 0.014, and 0.016, respectively). Tumor biology, including estrogen and progesterone receptors, and molecular subtypes showed association with the LN+ group (*p* < 0.001; *p* = 0.001; and *p* = 0.001, respectively). A total of 117 (73.6%) patients were eligible for the SOUND study and the potential operating room time saved was 2696.81 min. **Conclusions:** De-escalating strategies may rationalize healthcare activities. Multifactorial risk stratification may further refine the selection of patients who could benefit from SLNB omission.

## 1. Introduction

In the past century, sentinel lymph node biopsy (SLNB) emerged as the standard of care in patients with early breast cancer (EBC). In milestone papers, Veronesi et al. and Karg et al. aimed to perform axillary surgical de-escalation in EBC, given the equivalent oncological outcome of SLNB compared with axillary lymph node dissection (ALND) [1,2]. While previously ALND was considered the gold standard, in both clinical trials, ALND with curative intent in EBC was preserved only for cases of SLN involvement. 

In 2017, the results of the 10-year American College of Surgeons Oncology Group Z0011 (ACOSOGZ0011) represented another milestone in the de-escalation of axillary surgical management. The ACOSOGZ0011 study demonstrated that even in cases of limited axillary disease in patients undergoing breast-conserving surgery (BCS), and medical and radiation adjuvant treatment, the omission of ALND did not lead to detrimental effects on locoregional and distant outcomes [3]. Once confirmed by other randomized clinical trials, the safe omission of ALND required new paradigms and strategies in locoregional axillary treatment [4,5,6,7]. 

In the modern era of breast surgery, while evidence from the ACOSOGZ0011 trial demonstrated reduced morbidity for a significant cohort of EBC patients, these results led to a reevaluation of the clinical significance of SLNB in the era of genomic assessment and consequently considerations of alternatives to surgical axillary staging [8]. From this perspective, the recent SOUND study aimed to demonstrate that a safe complete de-escalation with axillary lymph node (ALN) ultrasound may be sufficient to control local and distant disease without affecting adjuvant treatment [9]. While the cutting-edge evidence from the SOUND study will be practice-changing for a significant proportion of patients, many authors have started to explore the limitations of the study and determine which will be the real population that will eventually benefit from this study [10,11]. In order to establish whether other patients may safely omit SLNB, a study named sentinel omission risk factor (SOFT) 1.23 was planned in our department. Therefore, the aim of the present paper is to identify any preoperative variables that may be applied to the selection of a wider population where axillary invasive assessment may be safely omitted, and evaluate the potential beneficial effect of de-escalating strategies on surgical room occupancy.

## 2. Materials and Methods

A single-center retrospective study from a prospectively maintained database, named SOFT 1.23, was planned. The primary endpoint of the study was to underline the preoperative prognostic factors related to lymph node (LN) and/or SLN metastasis, aiming at surgical de-escalation. The institutional ethical committee Area Territoriale Lazio 2 approved the study (n° 122/23). Therefore, all consecutive patients fulfilling the inclusion criteria were enrolled between 1 January 2022 and 30 June 2023. Secondary outcomes included the analysis of surgical room occupancy for SLNB in patients fulfilling the inclusion criteria for the SOUND study population. 

### 2.1. Patients’ Characteristics

All consecutive invasive EBC patients (cT1a-2 cN0 cM0) scheduled for BCS and SLNB were included in the study. Additional inclusion criteria were age ≥ 18 years, undergoing an upfront surgical procedure without prior primary medical treatment. All patients enrolled prior to surgery required a clinical examination, bilateral mammogram, breast and axillary ultrasound, and histological breast cancer (BC) diagnosis (triple assessment). For patients under the age of 40 years, a mammogram was not considered mandatory, and core needle biopsy (CNB) or vacuum-assisted biopsy (VAB) were considered suitable for preoperative BC diagnosis. Patients who underwent diagnostic lumpectomy were excluded from the study. Bilateral contrast enhancement magnetic resonance or bilateral contrast enhancement mammogram were not considered mandatory for preoperative assessment. Moreover, patients with distant metastasis, a medical history of thoracic radiotherapy, ipsilateral breast or axillary surgical treatment that could interfere with ALN appearance, and pregnancy were excluded from the analysis. Before admission to our facility, all patients signed a general study consent for enrollment in our database. Moreover, once included in the study, all patients provided a specific written consent for the study. 

### 2.2. Data Collection 

A retrospective analysis of our prospectively maintained database was performed. Demographic data analyzed in the study included age, age at menarche, age at diagnosis, and body mass index (BMI). BC diameter was retrieved from the radiological reports of ultrasound and mammogram. The clinical stage was assessed according to the AJCC 2018 eighth edition of the TNM classification with clinical examination, bilateral mammogram, breast and axillary ultrasound, and histological BC diagnosis [12]. BC preoperative characteristics regarding tumor dimensions and biomolecular features were included in the analysis, including histotype, estrogen receptor (ER), progesterone receptor (PR), Ki67, and epidermal growth factor receptor-2 (HER2) score according to the ASCO HER-2 2018 guideline update [13]. Further analysis included tumor grade according to the Nottingham histologic score system (the Elston–Ellis modification of Scarff–Bloom–Richardson grading system), and biomolecular classification according to the 2013 San Gallen criteria [14]. Intraoperative data were retrieved from surgical notes and single surgical procedures were calculated separately (breast-conserving surgery and SLNB). Due to internal policy, SLNB frozen sections were performed in the case of clinical suspicion, when SLN were enlarged >1 cm, or when clinical involvement was suspected during surgery. Intraoperative staining and definitive staining were assessed for pN. 

### 2.3. Statistical Analysis 

All data were collected in a prospectively maintained database (Excel 2016 ver 2406, Microsoft 365, Washington, DC, USA). Statistical analysis was conducted using the statistical package for the social sciences (SPSS v.15.0; SPSS, Inc., Chicago, IL, USA). Patients were grouped based on ALN involvement according to the definitive pNx(sn) stage (pN0(sn) for LN− and pN1(sn) for LN+). Continuous variables were expressed as medians and interquartile ranges (IQR), while categorical variables were presented as frequencies and percentages. Continuous variables between groups were compared using the Student’s *t*-test or Mann–Whitney U test, based on the Kolmogorov–Smirnov test results. Categorical and dichotomous variables were compared using the chi-square test or Fisher’s exact test, depending on the sample size. For multiple categorical variables, Monte Carlo correction was applied to both tests (e.g., T stage, biomolecular classification). Variables with *p* < 0.05 in the univariate analysis were considered statistically significant.

## 3. Results

**Figure 1:**
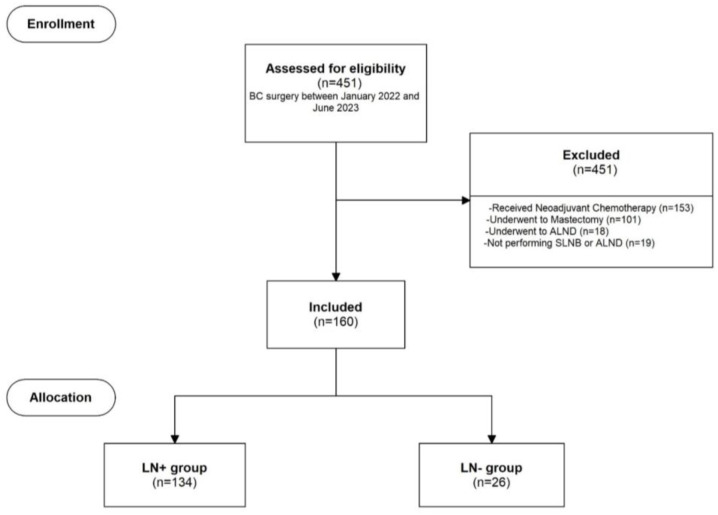
Study flowchart. BC: breast cancer; ALND: axillary lymph node dissection; SLNB: sentinel lymph node biopsy.

Between January 2022 and June 2023, 451 invasive BC patients underwent breast surgery in our department. A total of 291 BC patients were excluded from the study for the following reasons: 153 received neoadjuvant chemotherapy, 101 underwent mastectomy, 18 underwent upfront ALND, and 19 did not undergo any axillary surgical exploration after multidisciplinary assessment due to age or low-performance status. Therefore, 160 patients were included in the study, as shown in Figure 1. The median age of the population was 61 (50;74) years. Among these, 26 patients (16.25%) had axillary macrometastatic disease identified during pathological assessment (LN+), while 134 patients (83.75%) were included in the LN− group.

The univariate analysis comparing LN+ and LN− groups is reported in Table 1. Preoperative data such as age (*p* = 0.880), BMI (*p* = 0.427), localization (quadrant) (*p* = 0.771), laterality (right vs. left) (*p* = 0.433), and BI-RADS breast density (*p* = 0.237) did not show a statistically significant association with LN status. Additionally, dividing the population by age, over 70 showed no statistically significant result (*p* = 0.768). Multifocality/multicentricity was associated with a higher rate of SLN involvement (20.90% vs. 42.31%; *p* = 0.020). Higher clinical stages were also associated with SLN involvement (*p* = 0.016). However, the histological assessment revealed that the preoperative tumor histology classification or tumor grade did not demonstrate a statistically significant difference between groups, with similar distribution even across rare variants (*p* = 0.103; *p* = 0.296, respectively).

In terms of tumor biology, a significantly higher rate of ER expression was observed in the LN+ group compared to the LN− group (90% vs. 74%; *p* < 0.001). Similar results were reported for PR expression (90% vs. 70%; *p* = 0.001). Conversely, Ki67, whether calculated as a continuous variable or as a dichotomous variable with cut-offs of 7.5% and 14%, did not show a statistically significant difference between the two groups (*p* = 0.756; *p* = 0.287; *p* = 0.687, respectively). Her2 expression patterns also showed no significant difference between the two groups (*p* = 0.865). Finally, both groups were analyzed according to the 2013 St. Gallen biomolecular classification. A higher rate of luminal B tumors was documented in the LN+ group compared to the LN− group, with fewer non-luminal tumors (3.85% vs. 13.44%) and luminal A tumors (30.77% vs. 55.22%) (*p* = 0.011).

Operating room occupancy analysis showed a median time per surgical procedure of 92.64 (58.39–141.34) minutes. Among the study population, 27 patients (16.86%) underwent intraoperative histological examination. In 3 out of these 27 patients (11.11%), the frozen section predicted LN involvement, and ALND was performed. The SLNB frozen section evaluation exhibited a 75% sensitivity and a 93% specificity (Table 2). The median axillary procedure time was 23.69 (16.87–27.57) minutes, while for conservative surgery without axillary surgery, the median duration was 70.60 (35.66–117.50) minutes. 

Finally, our sample was stratified based on the SOUND study criteria to evaluate the number of SLNB procedures that could have been avoided. A sub-analysis was conducted on the population with lesions < 2 cm in preoperative assessment. Among them, a total of 117 (73.6%) patients were deemed eligible for the SOUND study. Within this subgroup, 11 (9.48%) BC patients showed an axillary positivity. The potential operating room time spared was 2696.81 min, which could have accommodated an additional 38.20 conservative surgery procedures without SLNB.

## 4. Discussion

In the last 30 years, the pursuit of safe axillary surgical de-escalation has been a pivotal research focus in modern breast surgery, aiming to improve the quality of life for patients with EBC. In this regard, the SOUND study has demonstrated for the first time that a complete and safe de-escalation is possible in a specific patient subgroup [9]. However, concerns have been raised by several authors regarding the lack of criteria related to age, menopausal status, or tumor biology for enrollment, which may limit its broad applicability [10]. Our real-world retrospective analysis demonstrated how factors such as ER and PR expression, and molecular subtype could be implemented in the de-escalation decision-making process.

Traditionally, the determination of pathologic nodal status obtained through surgical exploration has been the main factor driving recommendations for adjuvant systemic therapy and radiation therapy. Without a deeper understanding of the biological pathways underlying BC progression, pathological nodal status was considered the strongest predictor of distant disease [15]. In this context, the SLNB framework in the 1990s represented the first successful attempt toward axillary de-escalation. It effectively discriminated between SLNB node-negative and node-positive patients, with the latter eventually undergoing ALND [16]. However, further evidence from the ACOSOG Z0011 and AMAROS trials demonstrated that even in cases of a low nodal disease burden, completion of ALND could be avoided in the context of multidisciplinary treatment [3,17]. In recent years, as the therapeutic significance of axillary surgery has diminished, molecular genomic assessment has become a standard of care in the breast cancer decision-making process. This approach can determine the benefit of chemotherapy even in node-positive postmenopausal women [18,19,20,21]. Moreover, in addition to genomic assessment, innovative adjuvant treatments, and advanced breast imaging strategies, the concerns have been shifted from oncological safety to identifying which patient subsets would benefit the most from axillary surgical de-escalation [22,23,24,25]. 

Currently, supported by the growing body of evidence supporting de-escalating protocols, only a few routine indications are maintained for ALND. However, while safe, a large number of clinical strategies still partially rely on ALN status [26]. For instance, according to the RxPONDER trial, premenopausal luminal patients with one to three positive SLNs should undergo chemotherapy regardless of their oncotype DX recurrence score [21]. Moreover, findings of the monarchE trial restricted the use of innovative treatments such as abemaciclib in patients with ≥4 positive ALNs or 1–3 positive ALNs plus other high-risk features [27].

Combining these results with the increasing evidence supporting surgical de-escalation in different clinical settings, a mere dimensional criterion outlined in the SOUND study may not include some patients who could potentially benefit from a non-invasive axillary assessment [26]. Table 3 provides similar studies published in the last 5 years.

Tumor size has been classically associated with ALN involvement in BC patients. Historical series demonstrate how tumor diameter represents one of the strongest factors associated with SLN metastasis [51,52]. As stated before, tumor dimension was considered as the first inclusion criterion for the SOUND study [9]. Our experiments corroborate previous results in which tumor dimension has been highly associated with SLN metastasis. Moreover, similar results were obtained in pN1(sn) between our series and the SLNB group from the SOUND study (9.58% vs. 8.6%) [9]. As expected, the higher rate observed in our series may be justified by multicentric/multifocal tumors that were included in our analysis but then excluded from the SOUND study. Our series confirmed the association between multicentricity/multifocality and an increased rate of SLNB metastasis. 

Additionally, our preliminary analysis demonstrated how SLNB may be predicted from well-known preoperative factors. Besides the SOUND results, age has been classically investigated in order to select a population for a safe de-escalation. Unlike the findings of the study conducted by Abdulla et al., our data did not demonstrate a difference in age distribution between groups [33]. Robust evidence demonstrated how older age (>70 years) may affect changes in hormonal receptor status and HER2 status. Moreover, older BC populations tend to present with smaller tumors with a low ki67 index, which could eventually affect the SLNB rate [33,53]. In line with our results, we believe that age should not be considered a discriminating factor a priori to avoid surgical axillary staging. Instead, it should be integrated into a multidisciplinary approach to assess whether axillary staging could influence adjuvant treatment and patients’ outcomes [54].

Besides tumor dimension and age group, modern multidisciplinary treatment encompasses tumor biological characteristics in order to tailor a multidisciplinary treatment for each patient [55]. In our analysis, we demonstrated how biological characteristics may predict SLNB results. Specifically, in the LN+ group a higher rate of ER, PR expression, high nuclear grade, and luminal B neoplasms was observed. PR and ER are key prognostic biomarkers, defining hormone-positive breast cancer and its response toward systemic hormonal therapy and/or innovative target therapy as cyclin-dependent kinase 4/6 inhibitors [56,57,58]. In clinical practice, PR-positive breast cancers show a higher endocrine response than PR-negative ones. While the predictive efficacy of endocrine therapy response is limited, PR remains a valuable indicator. Its role in regulating genes in a cell cycle-dependent manner suggests that some key PR target genes, especially those in the S phase, might have been missed in studies using unsynchronized cell lines [56,57,58]. Moreover, ER and PR status are routinely incorporated into the classification of intrinsic breast cancer subtypes [14]. 

Intrinsic BC subtypes are used to drive multidisciplinary treatment in association with performance status and clinical stage. Besides their clinical application in multidisciplinary treatment, some authors have started to explore their clinical application in SLNB status prediction in order to reduce the potential harm of disease underestimation or overtreatment [59]. As is widely known, due to the novel neoadjuvant strategies and preferential hematogenous spread of triple negative and HER2 type BC, LN involvement rates were lower compared to luminal tumors. Therefore, it supports potential biological-driven safe axillary de-escalation strategies in clinical settings different from those of the SOUND study [59,60,61,62,63]. As expected, luminal B tumors in our series were associated with higher rates of SLN metastasis, which shares with luminal A its indolent counterpart, a specific lymphotropic spread. 

We are aware that our study may have some limitations. Due to its exploratory nature, no power analysis and multivariate analysis were performed. Moreover, retrospective analysis may have altered our results. Despite this potential limitation, the monocentric design provides a greater homogeneity among populations. Despite the mentioned limitations, this preliminary analysis has allowed us to identify factors associated with SLNB involvement such as tumor size ultrasound and biomolecular characteristics such as grade, luminal subtype, and ER/PR expression. Further, larger studies are needed to assess the role of each single risk factor to predict SLNB involvement in a multidisciplinary setting and to promote a rationalized approach useful to provide a safe de-escalating approach in different settings where clinicopathological factors, biological behavior, and genomic assessment may reduce the impact on patient quality of life and healthcare costs [64,65,66]. In our series, 73.6% of BC patients were eligible for the SOUND study, allowing a further 38.20 conservative surgery procedures to be performed without SLNB. In this clinical setting, without a clear benefit for the patients, the lack of implementation of de-escalation protocols could lead to multiple adverse effects such as detrimental effects on quality of life, increased surgical complications, escalating healthcare costs, and even a rise in healthcare-related environmental pollution [65,66,67,68].

In conclusion, our study demonstrated that applying the results of the SOUND study can effectively rationalize healthcare activities, potentially improving patient quality of life and reducing the detrimental effects of invasive ALN staging. While preliminary results from a large database demonstrated how short-term outcomes may be affected by invasive ALN staging, a comparative study to assess patients’ reported outcome measures (PROM) or quality of life is lacking, and further studies are needed to explore PROM in axillary EBC staging. 

Regardless of these limitations, in the future, we believe that risk stratification of the population, beyond mere size criteria, through known preoperative prognostic and predictive factors, could further refine the selection of patients who could benefit from de-escalating strategies.

## Figures and Tables

**Table 1:** Demographic and preoperative data.

	LN− Group (n = 134)	LN+ Group (n = 26)	*p*-Value
**Age (IQR) years**	60 (50; 71)	67 (50; 74)	0.880
**Age > 70 years yes, n (%)**	36 (26.87%)	8 (30.77%)	0.768
**BMI (IQR) kg/m^2^**	24.22 (22.06; 27.31)	26,04 (23.38; 28.52)	0.427
**Localization (quadrant), n (%)**			
Upper-outer (UOQ)	57 (42.55%)	11 (42.31%)	0.771
Upper-inner (UIQ)	15 (11.19%)	4 (15.38%)	
Lower-outer (LOQ)	18 (13.43%)	4 (15.38%)	
Lower-inner (LIQ)	21 (15.67%)	5 (19.23%)	
Other localization	23 (17.16%)	2 (7.69%)	
**Laterality (right vs. left), n (%)**			
Right	69 (51.49%)	11 (42.31%)	0.433
Left	65 (48.51%)	15 (57.69%)	
**Focality (unifocal vs. multifocal), n (%)**			
Unifocal	106 (79.10%)	15 (57.69%)	0.103
Multifocal	14 (10.45%)	6 (23.08%)	
Multicentric	14 (10.45%)	5 (19.23%)	
Unifocal	106 (79.10%)	15 (57.69%)	0.020 *
Multifocal/multicentric	28 (20.90%)	11 (42.31%)	
**Clinical Stage, n (%)**			
cT1a	9 (6.72%)	1 (3.85%)	0.014 *
cT1b	44 (32.84%)	5 (19.23%)	
cT1c	58 (43.28%)	8 (30.77%)	
cT2	23 (17.16%)	12 (46.15%)	
**Breast Density, n (%)**			
A	6 (4.48%)	7 (26.92%)	0.237
B	99 (73.88%)	14 (53.85%)	
C	23 (17.16%)	4 (15.38%)	
D	6 (4.48%)	1 (3.85%)	
**Tumor Diameter, mm (IQR)**	12 (9.0; 18.5)	19 (13.0; 23.5)	0.016 *
Tumor size > 20 mm yes, n (%)	23 (17.16%)	12 (46.15%)	0.001 *
Microcalcification yes, n (%)	31 (23.13%)	9 (34.62%)	0.184
**Tumor Histology, n (%)**			
Invasive ductal	110 (82.09%)	19 (73.08%)	0.103
Invasive lobular	17 (12.69%)	4 (15.38%)	
Other	7 (5.22%)	3 (11.54%)	
**Tumor Grade, n (%)**			
1	17 (12.69%)	4 (15.38%)	0.296
2	85 (63.43%)	17 (65.39%)	
3	30 (22.39%)	2 (7.69%)	
N/A	2 (1.49%)	3 (11.54%)	
**Immunohistochemistry**			
**ER % (IQR)**	74 (72.5; 95)	90 (80; 95)	<0.001 *
**PR % (IQR)**	70 (1.25; 90)	90 (40; 95)	0.001 *
**Ki67 % (IQR)**	18 (10; 30)	25 (16.75; 30)	0.756
**Ki67 > 7.5% yes, n (%)**	110 (82.09%)	19 (73.08%)	0.287
**Ki67 > 14% yes, n (%)**	82 (61.19%)	17 (65.38%)	0.687
**HER2 Score, n (%)**			
0	31 (23.14%)	4 (15.38%)	0.865
1	83 (61.94%)	15 (57.69%)	
2	7 (5.22%)	3 (11.55%)	
3	13 (9.70%)	4 (15.38%)	
**Molecular Subtype, n (%)**			
Luminal A	**74 (55.22%)**	**8 (30.77%)**	0.011 *
Luminal B	**42 (31.34%)**	**17 (65.38%)**	
HER2 type	**4 (2.99%)**	**1 (3.85%)**	
Triple negative	**14 (10.45%)**	**0 (0%)**	

All continuous variables are expressed as median and interquartile range (IQR), while categorical variables are indicated as frequencies and percentages. Continuous variables between groups were compared with a Student *t*-test or Mann–Whitney U test, according to the Kolmogorov–Smirnov test. Categorical and dichotomous variables were compared between groups with a chi-square test or Fisher’s exact test according to the sample size. *p*-values < 0.05 are highlighted with * and considered statistically significant. BMI: body mass index; ER: estrogen receptor; LN: lymph node; PR: progesterone receptor.

**Table 2:** SLNB Frozen section performance according to the SLNB definitive staining.

		SLNB Definitive Staining Macrometastasis		
		Yes	No	Total
**SLNB Frozen Section** **Macrometastasis**	**Yes**	3 (11.54%)	0 (%)	3 (11.54%)
	**No**	1 (3.84%)	22 (84.62%)	23 (88.46%)
	**Total**	4 (15.38%)	22 (84.62%)	26 (100%)

SLNB: sentinel lymph node biopsy.

**Table 3:** Trial investigating SLNB positive predictive value in EBC.

Author (Year)	Country	Design	Clinical Stage	n=	ALN+ n=	Variables Examined	Univariate Preoperative Prognostic Factor	Multivariate Preoperative Prognostic Factor
Akbari et al. [28] (2024)	Iran	Retrospective	cT1-3 cN0	73	33	Clinical Pathological	LVI Histology Ki67	N.A.
Lee et al. [29] (2024)	South Korea	Retrospective	pT1(mi) cN0	1688	70	Clinical Pathological	Age Breast surgery Axillary surgery SLNs number Tumor size Grade LVI Foci of microinvasion number ER PR Molecular subtype Radiotherapy Endocrine therapy	Age Axillary surgery SLNs number LVI Foci of microinvasion number ER PR
Liu et al. [30] (2024)	China	SEER database	BCS cT1-3 cN0	16983	2338	Clinical Pathological	Age Race Tumor site Tumor size grade ER PR SLN number Radiation Chemotherapy	Age Race Grade Radiation ER PR
Pang et al. [31] (2024)	China	Retrospective	cT1-2 cN0	118	N.A.	US CEUS Clinical Pathological	Age HER2 Nutrient vessel BC CEUS enhancement pattern SLN CEUS patterns	CEUS pattern of enhancement lesion CEUS patterns of SLN
Zhang et al. [32] (2024)	China	Retrospective	cT1-2 cN0	998	228	US Clinical Pathological	LVI Tumor location ALN US Tumor size Histological grade	Lymphovascular invasion ALN US Maximum diameter
Abdulla et al. [33] (2023)	Bahrain	Retrospective	cT1-3 cN0	160	35	Clinical Pathological	Age Grade ER LVI Tumor size	Tumor grade ER LVI Tumor size
Jin et al. [34] (2023)	China	SEER research plus data	TNBC cT1-3 cN0	17554	N.A.	Clinical Pathological	Age Race Histology Grade Tumor size Marital status Sex	Age Race Tumor size Grade
Fu et al. [35] (2022)	China	Retrospective	cT1-2 cN0	141	26	Clinical Pathological	Tumor location ER PR LVI	Tumor location PR LVI
Gao et al. [36] (2022)	China	SEER database	cT1-3 cN0 ALN− vs. ALN+(1–2) vs. ALN+ (>2)	4753	ALN+(1-2) = 1961) ALN + (>2) = 371	Clinical Pathological	ALN− vs. ALN+(1–2) vs. ALN+ (>2): Age Race Tumor size Tumor location Molecular subtype ER PR HER2 Grade Histology	ALN− vs. ALN+: Tumor size Tumor location Molecular subtype Grade Histology
Wu et al. [37] (2022)	China	SEER database	MBC cT1-3 cN0	665	51	Clinical Pathological	Age Tumor size Grade HER2	Age ≥ 70 Tumor size Grade II/IV
Xiong et al. [38] (2022)	China	Retrospective	cT1-2 cN0	1076	437	US Clinical Pathological	Age Grade Multifocality Molecular subtype Tumor location Tumor size US margin Skin distance	Age Grade Molecular subtype Tumor location Tumor size US margin Skin distance
Yiming et al. [39] (2022)	China	Retrospective	cT1-3 cN0	99	49	US Clinical Pathological	Age Tumor size CK5/6 HER2 TP53 mutation BRCA1 mutation BRCA2 mutation	N.A.
Zhu et al. [40] (2022)	China	prospective	cT1-2 cN0	114	59	US CEUS Clinical Pathological	Multifocal HER2 Tumor size Resistance index CEUS extended range	Tumor diameter HER2 Tumor size Resistance index
Hu et al. [41] (2021)	China	Retrospective	c T1-3 cN0.	624	147	US Clinical Pathological	Age BMI Her2 type vs. LumA Ki67 Tumor size Inner echo Calcification Color Doppler flow Aspect ratio	Age BMI Ki67 Tumor size Tumor margin Calcification Aspect ratio
Minami et al. [42] (2021)	Japan	Retrospective	c T1-2 cN0.	313	54	Clinical Serum Pathological	IGT Tumor size Nipple distance Tumor location Tumor stage	IGT Tumor size Nipple distance
Wang et al. [43] (2021)	China	Retrospective	c T1-3 cN0.	297	74	US CEUS MAM Clinical Pathological	BMI Tumor resection biopsy Tumor size LVI ER PR CK5/6 HER2 ALN US aspect ratio US lymphatic structure Cortical medulla US SLN CEUS patterns SLN CEUS aspect ratio ALN MAM aspect ratio	BMI SLN US aspect ratio SLN CEUS patterns SLN CEUS aspect ratio ALN MAM aspect ratio CK5/6
Yang et al. [44] (2021)	China	Retrospective	cT1 cN0	154	32	Clinical Serum Pathological	Tumor size LVI PLR NLR	N.A.
Catteau et al. [45] (2020)	Belgium	Retrospective	Tis-T4	212		Clinical Pathological	Age Tumor size T stage T grade LVI Molecular classification Ki67 20%	N.A.
Fan et al. [46] (2020)	China	Retrospective	cT1-2 cN0	121	56	Clinical Serum Pathological	CA153 CEA WBC Tumor size ER PR	CA153 CEA WBC
Fan et al. [47] (2020)	USA	National cancer database	cT1mi cN0	2609	76	Clinical Serum Pathological	N.A.	Grade Age Molecular subtype
He et al. [48] (2020)	China	Retrospective	cT1-2 cN0	556	235	Clinical Pathological	SLN positive absolute number SLN metastasis rate LVI	SLN positive absolute number SLN metastasis rate LVI
Takada et al. [49] (2020)	Japan	Retrospective	cT1 cN0	332	16	Clinical Pathological	Tumor size LVI TILS yes/no	Tumor Size LVI TILS yes/no
Zhang et al. [50] (2019)	China	Retrospective	cT1-2 cN0	1671	541	US Clinical Pathological	Tumor size Palpable Nipple distance Tumor location Grade ISAT IIAT	Tumor size Nipple distance ISAT IIAT

ALN: Axillary lymph nodes, BC: breast cancer; BCS: breast-conserving surgery; BMI: body mass index; BRCA: breast cancer gene; CA153: cancer antigen 15-3; CEA: carcino-embryonic antigen; CEUS: contrast enhancement ultrasound; CK5/6: cytokeratin 5/6; EBC: early breast cancer; ER: estrogen receptor; IGT: impaired glucose tolerance; ISAT: infiltration of subcutaneous adipose tissue; IIAT: infiltration of the interstitial adipose tissue; LVI: lymphovascular invasion; HER2: human epidermal growth factor receptor 2; MAM: mammogram; MBC: mucinous breast cancer; PR: progesterone receptor; SLN: sentinel lymph node; SLNB: sentinel lymph node biopsy; TNBC: triple negative breast cancer; TP53: tumor protein 53.

## Data Availability

The raw data supporting the conclusions of this article will be made available by the authors upon request.
